# Evaluation of Soil Management Effect on Crop Productivity and Vegetation Indices Accuracy in Mediterranean Cereal-Based Cropping Systems †

**DOI:** 10.3390/s20123383

**Published:** 2020-06-15

**Authors:** Roberto Orsini, Marco Fiorentini, Stefano Zenobi

**Affiliations:** Department of Agricultural, Food and Environmental Sciences (D3A), Agronomy and Crop Science Section, Marche Polytechnic University, 60131 Ancona, Italy; m.fiorentini@univpm.it (M.F.); s.zenobi@univpm.it (S.Z.)

**Keywords:** durum wheat, nutritional status, soil organic matter, no tillage, conventional tillage, remote sensing, multispectral imagery

## Abstract

Mostly, precision agriculture applications include the acquisition and elaboration of images, and it is fundamental to understand how farmers’ practices, such as soil management, affect those images and relate to the vegetation index. We investigated how long-term conservation agriculture practices, in comparison with conventional practices, can affect the yield components and the accuracy of five vegetation indexes. The experimental site is a part of a long-term experiment established in 1994 and is still ongoing that consists of a rainfed 2-year rotation with durum wheat and maize, where two unfertilized soil managements were repeated in the same plots every year. This study shows the superiority of no tillage over conventional tillage for both nutritional and productive aspects on durum wheat. The soil management affects the vegetation indexes’ accuracy, which is related to the nitrogen nutrition status. No-tillage management, which is characterized by a higher content of soil organic matter and nitrogen availability into the soil, allows obtaining a higher accuracy than the conventional tillage. So, the users of multispectral cameras for precision agriculture applications must take into account the soil management, organic matter, and nitrogen content.

## 1. Introduction

Providing a sufficient amount of food to satisfy the nutritional demand of the current population is the essential goal of global agriculture. By 2050, the global population is estimated to reach 2.6 billion people [1], so food production must increase by at least 70% before 2050 to support continued population growth [2]. In modern agriculture, conventional tillage (CT) techniques have allowed the adoption of crops, especially on large surfaces ensuring high yields: the mixing of surface horizons in preparing the seedbed allows the stabilization of the main crop to the detriment of the weed competitors. However, this intensification of the crops, although necessary for responding to the food needs of the growing demographic pressure, is proving unsustainable: in fact, the increment of soil erosion [3,4], the use of water, energy, and fertilizers, the disruption of soil structure, and the reduction of water use efficiency [5] will probably increase the environmental and economic pressures posed by intensified agricultural activities [6]; therefore, the negative consequences for the environment are evident [7,8,9].

To lower the pressure of pollution and costs, agricultural conservation practices are gaining worldwide popularity for their ability to optimize productivity and reduce the impact on the land’s natural resources [10].

In fact, reduced tillage and even no tillage (NT) bring benefits to the environment in terms of reduction of soil erosion, leaching of nitrates, reduction in the use of agricultural machinery, as well as a lower emission of greenhouse gases and fuel costs [11].

Furthermore, the low soil disturbance, with the addition of crop residues, increases the levels of humidity [12] and nutrients in the horizons of soil explored from the roots and the soil organic carbon [13,14], and it reduces the mineralization rate of the organic matter, nitrogen losses, and the soil erosion [15], so it’s possible sustain long-term crop production [16,17,18].

These economic and environmental benefits underpin the three pillars of conservation agriculture (CA) such as NT, the adoption of crop rotations, and in-situ residue conservation and permanent soil cover [19].

Conservation practices are being studied on winter cereals, which are dominant crops in the Mediterranean semi-arid climate regions [20] where climate change is putting cereal yields at risk [21,22,23], and they are often penalized by extreme events such as long periods of extreme dryness alternated with a short heavy rainfall time. In the Mediterranean area, crop production can be improved with the adoption of CA techniques [10] and with the application of the right dose of nitrogen through the site-specific application of fertilizers [24].

To understand the phenological status and the soil during crop cycles, manual measurements of agronomic characteristics are necessary, but they are so labor intensive and time consuming [10]. As a solution, a modern farming management concept that responds to such challenge is Precision Agriculture (PA) [25,26], providing spatial and temporal data on the agricultural fields in a fast and economic way [27].

In fact, its remote sensing technology offers a more efficient way to obtain the large-scale mapping of plant parameters: the development of this technology is expected to increase the effectiveness of PA [28]. In particular, studies indicated that space-borne sensors can be used to obtain spatially extensive information from landscape at the global scale [29,30,31,32,33].

Using multispectral images collected by satellite, traditional aircraft, and unmanned aerial vehicles (UAVs), several studies [34,35,36,37] have examined vegetative conditions in agriculture.

In precision farming, UAVs are very widespread and are provided with multispectral cameras that measure different wavelength bands within visible and near infrared regions of the spectrum, which allow the formulation of a wide range of vegetation indices (VIs) informing on biomass [38,39], leaf area index [40,41], pigment content [42,43,44], nitrogen content [45,46], photosynthetic efficiency [47], water status [47,48], and cover (ground and residue) [49].

The contribution of spatial information technologies [50,51] defines site-specific management units (SSMU) that are useful for understanding the spatial variations of the crop, especially in terms of yield [52]. These variations are influenced by a multitude of factors including topographic, edaphic, biological, meteorological, and anthropogenic factors [53].

Climate change, as already mentioned, contributes to influencing this variability: in fact, in the Mediterranean area, there is a decrease in rainfall which, for example, influences the food activity of the microbial component of the soil [54,55], so it will be necessary to understand through the technology offered by PA the changes that take place in crop systems.

However, several studies show that this variability makes it complicated to use precision farming tools in and so often it is rather difficult to adapt them in farms that have to make lesser decisions [56,57,58]. As a consequence, precision farming technologies require support structures to facilitate learning and the reduction of uncertainty in the implementation and adaptation process [59,60].

The uncertainties detected with the instrumentation and the climate variability [54,61,62] join the information lack related to the evaluation of the soil management (SM) effect on the crop nutritional status and productivity through multispectral imagery.

Only recently [10] was it reported that the canopy height, cover, volume, and the Normalized Difference Vegetation Index (NDVI) calculated on cotton growth under NT was statistically higher than the cotton grown under CT. This suggests that soil management can influence not only the crop growth development, but also the NDVI values. The aim of this study is to describe the effect of different SM (NT versus CT) on the unfertilized durum wheat crop parameters, nutritional status, and VIs accuracy in order to draw up vegetation maps that are useful for the correct management of soil fertility and cropping systems productivity.

## 2. Materials and Methods

### 2.1. Experimental Site

**Figure 1:**
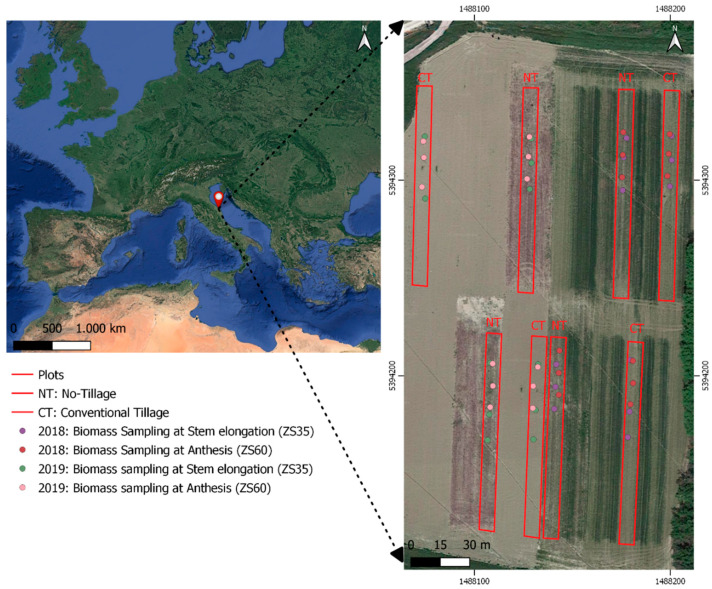
Experimental location (on the left), planimetry, and relative georeferenced positions of sampling biomass points during the two years experimental survey.

The experimental site is located at the “Pasquale Rosati” experimental farm of the Polytechnic University of Marche in Agugliano, Italy (43°32′ N,13°22′ E, at an altitude of 100 m above sea level and a slope gradient of 10%), on a silty-clay soil classified as Calcaric Gleyic Cambisols [63] (Figure 1).

The climate of the site is Mediterranean, on which was recorded a total rainfall of 801 mm between October 2017 and July 2018, while a contraction of 30% of rainfall was recorded during October 2018–July 2019 with a 560.8 mm of rainfall (Table 1).

In order to better represent the water dynamics into the soil–crop system, we estimated the monthly soil water balance (SWB) by using the following formulas (Equations (1) and (2)):

(1) SWB=P−ETc

(2) ETc=ETo (Hargraves)∗Kc (FAO)

where

P: monthly precipitation (mm);
ETc: monthly crop evapotranspiration (mm);
ETo: reference evapotranspiration calculated with the Hargraves formula (mm) [64];
Kc: crop coefficient [65]

The soil water balance calculated during the 2017–2018 growing season was 230 mm higher than the 2018–2019 growing season (Table 1) with a marked difference in the February–March period. The average minimum air temperature was higher on October 2017–July 2018 than October 2018–July 2019 with values respectively of 10 °C and 9.7 °C. Otherwise, the average maximum air temperature was higher on October 2018–July 2019 than October 2017–July 2018 with values respectively of 18.2 °C and 17.9 °C.

Soil properties in compared experimental plots are indicated in Table 2. Soil sampling was made with a Hand Huger (mod. Suelo HA-3) immediately before sowing. From each subplot, 3 samples were taken for a total of 12 soil samples analyzed for each year.

### 2.2. Experimental Design and Crop Management

The experimental site is a part of a long-term experiment established in 1994 and is still ongoing [66] consisting of a rainfed 2 years rotation with durum wheat (Triticum turgidum L. var. durum cv. Grazia, ISEA) in rotation with maize (Zea Mays L., DK440 hybrid Dekalb Monsanto, FAO Class 300) [67].

Within each field, two soil management techniques (main plot, 1500 m^2^) were repeated in the same plots every year and arranged according to a split plot experimental design with two replications. The conventional tillage (CT), which is representative of the business as usual tillage practice in the study area, was ploughed along the maximum slope every year by a moldboard (with 2 plows) at a depth of 40 cm in autumn. The seedbed was prepared with harrowing before the sowing date. The no-tillage (NT) soil was left undisturbed and was sprayed with herbicides before sowing prior to direct seed drilling. In this study, we will examine the unfertilized plots in order to describe the effect of different soil management techniques on the durum wheat crop parameters and on the crop nutritional status through the vegetation indices (VIs) computation. The dates (dd/mm/yy) of all the agronomic practices are reported in Table 3.

### 2.3. Measurements

At stem elongation and anthesis phenological stages (ZS35 and ZS60 respectively were ZS = Zadoks Scale [68]), we have measured crop parameters such as dry matter (g) and nitrogen (N) content (% and g m^−2^), and we have acquired multispectral images (MAIA S-2 multispectral camera) by using a UAV platform (DJI Matrice 600 pro) in order to compute the VIs algorithm. At crop maturity (ZS92), we measured the typical agronomic measurements, number of kernels per spike (KS), thousand kernel weight (TKW), and the grain yield (t ha^−1^) for both years under analysis in order to characterize the yield of the different soil management techniques.

#### 2.3.1. Crop Parameters

For each plot, we have randomly selected three test areas (Figure 1). At each test area, we have manually cut and collected fresh plants biomass in a georeferenced 0.5 m long-row using the GNSS HiPer HR receiver (Topcon, Ancona, Italy) for a total of 48 ground control points (GCPs).

The fresh plant biomass was oven-dried at 80 °C for 48 h and then, we weighed the dry biomass (g). Before analyzing for total N content, we ground the dry biomass to pass a 0.5 mm.

The N content (%) was determined by automated combustion analysis Dumas method [69,70] in an oxygen-enriched atmosphere at a high temperature (EA 1110 LECO CHNS-0 analyzer, Leco Corporation, St. Joseph, MI) in order to ensure complete combustion of the whole sample.

Starting from the N content (%) results, we calculated the N content (g m^−2^) by using the following formula (Equation (3)):

(3) $$gN\;m^{-2}=\frac{\frac{N\%\;*\;dry\;matter\;\left(g\right)}{100}}{0.085\;m^{2}}.$$

#### 2.3.2. Yield Components

In order to characterize the yield obtained by the compared treatments, we measured at crop maturity (ZS92) the number of KS, the thousand kernels weight (TKW), and the grain yield (t ha^−1^).

The KS and the TKW were estimated on 30 spikes randomly collected per plot. The grain yield (t ha^−1^) expressed in dry matter was measured by using a laboratory thresher for the three test areas (1 m long-row) per plot.

#### 2.3.3. Image Acquisition Processing

To generate the orthomosaic reflectance maps, we followed a process consisting of three steps: alignment and mosaicking of raw multispectral images, point cloud and mesh generation, and orthomosaic map export. For the first and third steps, we used the Pix4Dmapper (Pix4D, Lausanne, Switzerland), which is based on the structure from motion (SfM) algorithm [71]. This allows us to generate the orthomosaic reflectance map from the raw multispectral images acquired by each flight. For the second step, we used the geographical reference recorded by the D-RTK GNSS module equipped on the UAV platform. The newly generated orthomosaic reflectance map has been imported in QGis 3.4.8, an open source Geographic Information System, which was the software we used to complete the remaining two main steps of the image processing.

To complete the second main step, we inserted on QGis the GCPs by using a csv file format with the data source manager tool, and then we created for each GCP a polygon shape file of 0.085 m^2^, which corresponded to the sampling surface.

While in order to select the most relevant vegetation index (VI) calculated starting from multispectral imagery for precision agriculture application in a conservation agriculture context, we compered five vegetation index categories according with Xue and Su [72]. The VIs analyzed in this study are reported in the following Table 4.

The VIs calculation was carried out by a “Raster calculator” of QGis 3.4.8, which allows performing calculations on the basis of existing raster pixel values, and the results are written to a new raster layer with a GDAL supported format. The extraction of the VIs values was performed by using the “zonal statistics plugin” of QGis 3.4.8 by using the polygon shape file created for each GCP.

### 2.4. Statistical Analysis

All statistical analysis was performed with R. To highlight the significant effect of soil management (SM), year (Y), and the SMxY factorial combination to all the crop parameters analyzed, we performed an analysis of variance (ANOVA) to a linear model generated by using the generalized least squares approach.

Before performing any statistical analysis to identify a significant difference between the two soil managements in analysis, we performed a Shapiro–Wilk W test to evaluate the normality of distribution. When the P-value of the Shapiro–Wilk W test was below 0.05, we assumed that the data are not normally distributed; otherwise, the data are considered normally distributed.

When data were normally distributed, we performed the Bartlett test, which is used to test if k samples are from populations with equal variances or not. If the *p* value of the Bartlett output test was below 0.05, we assumed that the k samples are not from populations with equal variances, and so we performed the Welch One-Way ANOVA to identify a significant difference between the treatments under study. When the *p* value of the Bartlett output test was greater than 0.05, we assumed that the k samples are from populations with equal variances, and so we performed the t-test independent samples (*p* value = 0.05) to identify significant differences between soil managements.

When data were not normally distributed, we performed the Levene test, which is used to check that variances are equal for all samples when your data come from a non-normal distribution. If the *p* value of the Levene test was below 0.05, we performed the Friedman Test to highlight the significant difference between the treatments under study. When the *p* value of the Levene test was higher than 0.05, we performed the Kruskal–Wallis test to identify a significant difference between the soil management techniques.

To evaluate if the soil management can affect the relationships between VIs and N content (g m^−2^), we performed a linear regression analysis that is used to identify the existence of significant relationships (*: *p* ≤ 0.05; **: *p* ≤ 0.01; ***: *p* ≤ 0.001). In addition, we reported the coefficient of determination (R2) and relative root mean square error (RMSE) for each relationship.

## 3. Results

### 3.1. Crop Parameters

The ANOVA shows that the year (Y) factor has significantly affected all the crop parameters analyzed, while the soil management (SM) factor has significantly affected the nitrogen (N) content variables (% and g m^−2^).

For the N content (%), the ANOVA shows a significant effect of the interaction of year per soil management (Y x SM) (Table 5).

The 2019 year showed a significantly higher mean value of dry matter (DM) (g) and both N content variables (% and g m^−2^) than 2018 (Table 6), with a difference of 9.60 g, 0.74 and 2.70 for DM and N content (% and g m^−2^) respectively.

The no tillage (NT) showed a significantly higher N content (% and g m^−2^) than conventional tillage (CT) for both years (Table 6), which was equal to +0.63 for N content (%) and +0.76 for N content (g m^−2^) in 2018, and equal to +0.17 for N content (%) and +1.34 for N content (g m^−2^) in 2019.

### 3.2. Yield Components

The ANOVA shows for both year (Y) and soil management (SM) factors a significant effect on the yield components. In detail, the Y factor significantly affects the number of kernels per spike (KS) and the thousand kernel weight (TKW); the SM factor significantly affects the KS and grain yield (t ha^−1^) (Table 7). No significant effect of Y x SM interaction was observed.

The 2019 year showed a significantly higher value on the KS (+7) and a significantly lower value on the TKW (−7.7 g) than 2018, while no significant difference was observed for the grain yield (t ha^−1^) in the two-year comparison (Table 8).

The NT leads to a significantly higher value of the KS and grain yield (t ha^−1^) than CT in both the years under study (Table 8). In 2018, the NT obtained higher values of approximately 46% and 48% respectively for KS and grain yield than CT. While in 2019, the NT obtained higher values of approximately 35% and 35% respectively for KS and grain yield than CT.

### 3.3. Relationship between Vis and N Content (g m*^−2^*)

In the growing season of 2018, the NT system showed an R^2^ value of 0.81 on average and root mean square error (RMSE) of 0.57 on average, while the CT system showed an R^2^ value of 0.31 on average and an RMSE of 0.58 on average. During the growing season of 2019, the NT system showed an R^2^ value of 0.69 on average and RMSE of 1.44 on average; the CT system showed an R^2^ value of 0.45 on average and an RMSE of 1.35 (Table 9).

The previous discussion can also be extended to each individual VI analyzed; in fact, the values of R^2^ are always higher in NT than in CT in both growing seasons (Table 9).

Considering the 2018 year, we observed that Modified Soil-adjusted Vegetation Index (MSAVI2) is the most accurate VI, which reported a R^2^ on the NT of 0.96 while for CT, the R^2^ was 0.70. For 2019, we observed that the Normalized Difference Red Edge Index (NDRE) was the most accurate VI, which reported an R^2^ on the NT system of 0.95 and an R^2^ of 0.76 on the CT.

The NDRE and MSAVI2 are the only VIs that show a significant relationship with N content (g m^−2^) for both soil managements in each year.

By evaluating the average R^2^ obtained for all the VIs analyzed for each year and soil managements, we reported that NDRE is the most accurate VI to be related with the N content (g m^−2^) with a mean R^2^ value of 0.80 (Table 9).

### 3.4. Vegetation Index Maps

**Figure 2:**
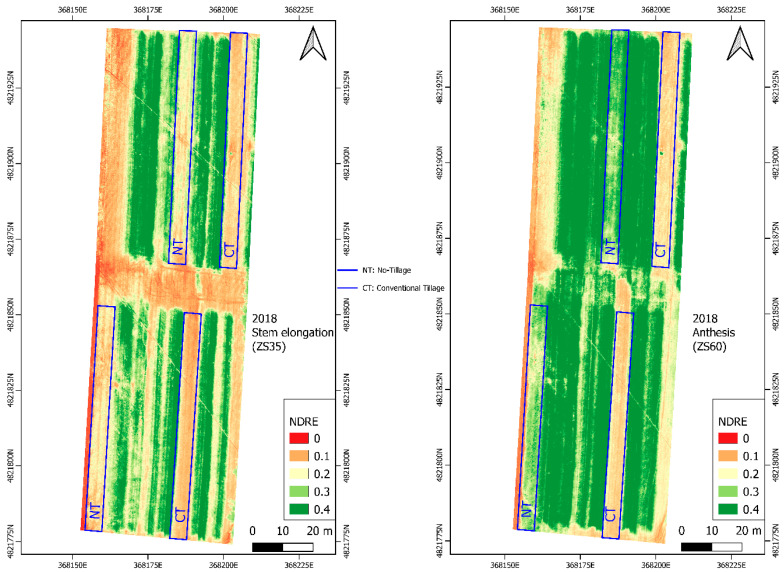
NDRE vegetation maps calculated at the stem elongation phenological stage (on the left) and at the anthesis phenological stage in the year 2018.

**Figure 3:**
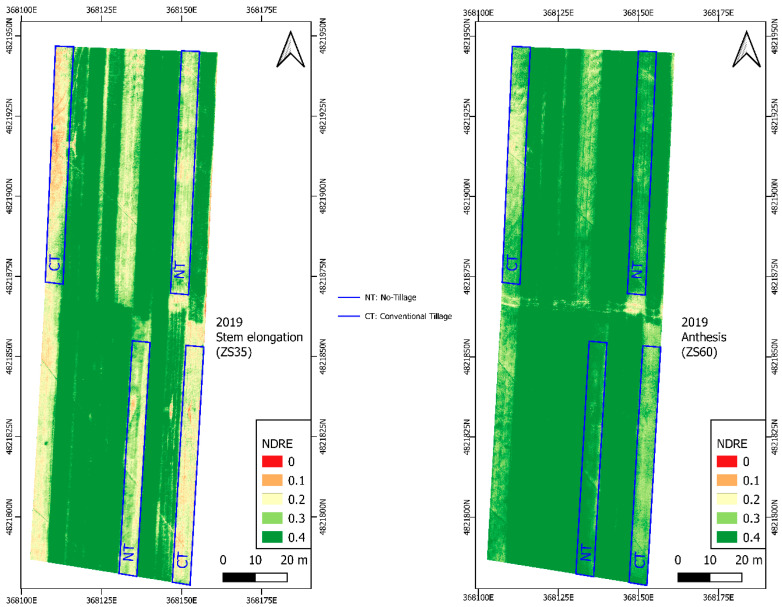
NDRE vegetation maps calculated at the stem elongation phenological stage (on the left) and at the anthesis phenological stage in the year 2019.

Figure 2 and Figure 3 show the NDRE vegetation maps corresponding to stem elongation (ZS 35 phenological stage) and anthesis (ZS 60 phenological stage) for both growing seasons (2018–2019) when the durum wheat reaches the maximum vegetative development.

The year 2019 showed a higher greenness than 2018 in each phenological stage; this is due to a significantly higher value of the DM (g) and N (% and g m^−2^) content (Table 6).

Within the same phenological stage, in the comparison between different years, NT showed significantly greater levels of greenness attributable, as previously mentioned, to the greater content of N (% and g m^−2^), KS, and grain yield (t ha^−1^) in both years under study (Figure 2 and Figure 3).

## 4. Discussion

The year (Y) factor showed a significant impact on DM (g), KS (n) and TKW (g) as reported on the same experimental site by Seddaiu et al., 2016 [67] and on both N content variables (%N and g m^−2^). These results show, as described from several authors [78,79], that the development of durum wheat during the season is strongly influenced by the climatic trend; in fact, the rainfall recorded in 2017–2018 growing season was 30% higher than the rainfall observed during the 2018–2019 (Table 1) season, and this probably led to a higher N leaching, which implies a reduction in the availability of N for the crop [80].

The probable N leaching occurring during the 2017–2018 growing season is confirmed by the monthly-estimated soil water balance (Table 1), which showed a difference of 230 mm with respect to the 2018–2019 growing season.

The annual difference is especially concentrated in the February–March period (154 mm and 111 mm respectively), so this indicates that during this period, some of the nitrogen that was made available for soil organic matter mineralization may have been leached. All these consequences are much more accentuated in the CT because it has a greater porosity of the soil than NT where there is an increased number of soil micropores that facilitate the storage of soil moisture [81,82,83], a lower soil organic matter than NT (Table 2) that plays a key role in water [84,85,86] and nutrient [87,88,89] retention also thanks to the mulching effect of the straw [88], as well as having no crop residues on the topsoil during the season due to the soil tillage, which involves a re-mixing of the horizons and consequently a dilution of the crop residues [90,91,92].

The year (Y) factor didn’t have a significant impact on the grain yield (t ha^−1^), this result could be induced by its two intrinsic variables such as KS and TKW (g), where we observed a dynamic balance.

In the 2018 growing season, the KS showed a lower value than the 2019 growing season, which implies a lower nutritional availability, due to the higher rainfall recorded, and therefore less fertility of the spike.

For TKW, we observed an inverse behavior; in fact, lower KS values correspond to higher TKW values as described also by Mohammadi et al., 2013 [93], who reported a significant Pearson correlation value of −0.52 between KS and TKW.

During June 2019, the period in which the milk and dough kernel development is occurring, the maximum and minimum air temperature were higher than June 2018 (2.4 °C and 1.7 °C respectively) (Table 1), this may have contributed to a greater loss of water from the caryopses with a consequent effect on the TKW reduction [94].

The soil management (SM) factor affected both N content variables (% and g m^−2^), KS, and grain yield (t ha^−1^) as reported also by Orsini et al., 2019b [95] and Fiorentini et al., 2019 [96].

The NT involve a number of other advantages with respect to CT, such as reduction of the management costs of the company [97,98,99], increased fertility of the soil, and positive effects on soil biochemical properties and biomass microbial [92,100,101,102], and this implies a stabilization of production in the medium to long term [103].

In contrast, the SM factor did not significantly affect the DM (g) and the TKW (g), confirming reports by De Vita et al., 2007 [104], according to which durum wheat grown at Vasto (Italy) did not show any significant difference in DM (g) and TKW for the years 2000 and 2001 for the Ct versus NT soil management analyzed.

The factorial combination of year and soil management (Y × SM) showed a significant effect (*p* ≤ 0.05%) on N content (%) as reported also by López-Bellido et al., 2013 [104].

Regarding the relationships between VIs and N content (g m^−2^), soil management shows a significant effect, as reported by Orsini et al. 2019a [66].

This may probably due to the greater amount of crop residues present on the NT system, which covers the soil surface, reducing soil disturbance [78] in the calculation of VIs starting from multispectral images.

Moreover, since the NT system is not disturbed by plowing, the residues of previous crops substantially increase water retention and consequently there is a greater availability of this element, thus determining greater crop development [5].

This dynamic is also confirmed by Ashapure et al. (2019) [10], who in cotton observed that the NT system, compared to the CT system, allows a significant increase on the NDVI (basic vegetation index category) in comparison with the CT system.

By evaluating the performance of the VIs to be related with the crop N content (g m^−2^), we suggest the use of NDRE and MSAVI2 to provide to farmers the vegetation index maps and the prescriptions maps for precision agriculture application.

## 5. Conclusions

The thermo-pluviometry trend strongly influences the development of durum wheat, both in yield and chemical composition.

This study shows the superiority of conservative agriculture over conventional agriculture for both nutritional and productive aspects on durum wheat.

We reported a dynamic balance on the yield components, in which KS and TKW are inversely proportional.

In addition, we confirmed again that the accuracy of VIs are related with the nitrogen nutrition status of durum wheat, and they also depend on the soil management. All the VIs analyzed obtained a higher accuracy in the NT system than in the CT system in both the years analyzed, which is due to the soil is not being disturbed by plowing and cultivation, previous crop residue substantially increasing water retention, and soil organic matter content contributing to higher plant growth and performance.

So, we advise to the potential users of multispectral images for precision agriculture application to take into account the soil management and related organic matter and nitrogen content into the soil.

In addition, we suggest the use of NDRE and MSAVI2 indices for durum wheat grown under a conservative agriculture context to provide vegetation maps and related prescription maps for the optimal monitoring of the nutritional status of durum wheat in Mediterranean agricultural contexts.

## Figures and Tables

**Table 1:** Thermo-pluviometric trend related to the durum wheat biological cycle during the experimental period.

Months	November	December	January	February	March	April	May	June	July	2017–2018
**Rainfall (mm)**										**Total**
2017–2018	124	96	29	173	143	37	95	48	57	802
2018–2019	42	61	70	22	36	59	165	1	105	561
Δ Rain	82	35	−41	151	107	−22	−70	47	−48	241
**Soil Water Balance (mm)**										**Total**
2017–2018	20	88	20	163	119	−44	−42	−8	−4	312
2018–2019	0	53	62	9	−4	−27	49	−51	−8	82
Δ Soil Water Balance	21	35	−42	154	123	−17	−91	43	4	229
**Min air T (°C)**										**Average**
2017–2018	7.9	4.1	5.2	2	5.7	11.8	14.9	17.7	20.5	10
2018–2019	9.3	3.7	2.4	4.5	7.4	9.1	11.7	19.1	20.3	9.7
Δ Min air T	−1.4	0.4	2.8	−2.5	−1.7	2.7	3.2	−1.4	0.2	0.3
**Max air T (°C)**										**Average**
2017–2018	11.1	11.9	12.8	8.3	13.3	21.5	23.7	27.8	30.8	17.9
2018–2019	11.9	11.1	9	13.2	17.7	18.6	20.3	30.5	31.5	18.2
Δ Max air T	−0.8	0.8	3.8	−4.9	−4.4	2.9	3.4	−2.7	−0.7	−0.3

**Table 2:** Soil properties of the 0–20 cm layer in the conventional tillage (CT) and no tillage (NT) unfertilized plots in 2019.

Soil Proprieties	SM ^1^	
	NT ^2^	CT ^3^
**Sand (g kg^−1^)**	127 (±21) a	120 (±19) a
**Silt (g kg^−1^)**	410 (±30) a	397 (±19) a
**Clay (g kg^−1^)**	463 (±36) a	483 (±22) a
**SOM ^4^ (g kg^−1^)**	18.0 (±2.8) a	13.2 (±2.1) b
**Total nitrogen (g kg^−1^)**	1.30 (±0.11) a	0.98 (±0.03) b

^1^ SM: soil management; ^2^ NT: no-tillage; ^3^ CT: conventional tillage; ^4^ SOM: soil organic matter. Within the same factor of variation, means that are followed by the same letter (a,b) are not significantly different at *p* < 0.05%.

**Table 3:** Agronomic management practices adopted during the two-year experimental period.

Agro-Technique	SM ^1^	2017–2018	2018–2019
Ploughing (40 cm)	CT ^2^	02/10/2017	26/09/2018
Weed control: Glyphosate ^3^	NT ^4^	30/10/2017	28/09/2018
Harrowing and seed bed preparation	CT	20/11/2017	01/10/2018
Sowing ^5^	All	21/11/2017	30/11/2018
Weed control: Pinoxaden ^6^	CT	28/03/2018	08/03/2019
Pest control: Azoxystrobin, Cyproconazole ^7^	All	24/04/2018	22/04/2019
Harvest	All	06/07/2018	07/07/2019

^1^ SM: soil management; ^2^ CT: conventional tillage; ^3^ dose: 2.25 kg ha^−1^ of active ingredient; ^4^ NT: no tillage; ^5^ Seed rate: 220 kg ha^−1^; row spacing: 0.17 m; ^6^ 30 g ha^−1^ of active ingredient; ^7^ dose: 0.16 l ha^−1^ of active ingredient.

**Table 4:** Agronomic management practices adopted during the two-year experimental period.

Vegetation Indices	Formula	References
ARVI ^1^	$\mathrm{ARVI}=\frac{\mathrm{NIR}-\;\mathrm{RB}}{\mathrm{NIR}+\mathrm{RB}}$ Where: RB= Red−y(Blu−Red)	Korhonen et al. (2015) [73]
MSAVI2 ^2^	$\mathrm{MSAVI}2=\frac{2\times \mathrm{NIR}+1-\sqrt{{\left(2\times \mathrm{NIR}+1\right)}^{2}-8\left(\mathrm{NIR}-\mathrm{Red}\right)}}{2}$	Leprieur et al. (2000) [74]
NDRE ^3^	$NDRE=\frac{NIR-Red\;Edge}{NIR+Red\;Edge}$	Barnes et al. (2000) [75]
VDVI ^4^	$\mathrm{VDVI}=\frac{2\times \mathrm{Green}-\mathrm{Red}-\mathrm{Blue}}{2\times \mathrm{Green}+\mathrm{Red}+\mathrm{Blue}}$	Wang et al. (2015) [76]
WDRVI ^5^	$\mathrm{WDRVI}=\frac{a\times \mathrm{NIR}-\mathrm{Red}}{a\times \mathrm{NIR}+\mathrm{Red}}$ Where: a=0.2	Gitelson (2004) [77]

^1^ ARVI: Atmospherically Resistant Vegetation Index; ^2^ MSAVI2: Modified Soil-adjusted Vegetation Index; ^3^ NDRE: Normalized Difference Red Edge Index; ^4^ VDVI: Visible-Band Difference Vegetation Index; ^5^ WDRVI: Wide Dynamic Range Vegetation Index.

**Table 5:** Results of the ANOVA applied to a linear model using generalized least squares for durum wheat.

Factor of Variation	df ^1^	DM ^2^	N Content	
		g	%	g m^−2^
Y ^3^	20	**	***	***
SM ^4^	20	n.s.	***	*
Y × SM	20	n.s.	*	n.s.

^1^ df: degree of freedom; ^2^ DM: Dry Matter; ^3^ Y: Year; ^4^ SM: Soil management; *: Significant at *p* < 0.05%; **: Significant at *p* < 0.01%; ***: Significant at *p* < 0.001%; n.s.: not significant.

**Table 6:** Durum wheat crop parameters analyzed during the growing seasons 2018 and 2019.

Year	SM ^1^	DM ^2^	N Content	
		g	%	g m^−2^
	NT ^3^	13.71 (±9.47) a	1.43 (±0.28) a	2.10 (±1.29) a
	CT ^4^	14.34 (±7.18) a	0.80 (±0.12) b	1.34 (±0.67) b
**2018**		**14.02 (±8.23) B**	**1.11 (±0.38) B**	**1.72 (±1.08) B**
	NT	26.92 (±18.95) a	1.93 (±0.51) a	5.09 (±2.71) a
	CT	20.32 (±13.53) a	1.76 (±0.35) b	3.75 (±2.02) b
**2019**		**23.62 (±16.45) A**	**1.85 (±0.44) A**	**4.42 (±2.44) A**

^1^ SM: soil management; ^2^ DM: dry matter; ^3^ NT: no-tillage; ^4^ CT: conventional tillage; means within columns that are followed by the same letter (lowercase letters for SM (a,b); uppercase letters for year (A,B)) are not significantly different at *p* < 0.05.

**Table 7:** Results of the ANOVA applied to a linear model using generalized least squares for durum wheat.

Factor of Variation	df ^1^	KS ^2^	TKW ^3^	Grain Yield
		n.	g	t ha^−1^
Y ^4^	20	***	***	n.s.
SM ^5^	20	***	n.s.	***
Y × SM	20	n.s.	n.s.	n.s.

^1^ df: degree of freedom; ^2^ KS: number of kernels per spike; ^3^ TKW: Thousand kernel weight; ^4^ Y: Year; ^5^ SM: Soil management; ***: Significant at *p* < 0.001%; n.s: not significant.

**Table 8:** Crop yield parameter measured at crop maturity on the 2018 and 2019 years.

Year	SM ^1^	KS ^2^	TKW ^3^	Grain Yield
			g	t ha^−1^
	NT ^4^	13 (±2) a	52.2 (±0.9) a	2.5 (±0.2) a
	CT ^5^	7 (±1) b	52.8 (±1.1) a	1.3 (±0.2) b
**2018**		**10 (±3) B**	**52.5 (±1.0) A**	**1.9 (±0.7) A**
	NT	20 (±2) a	44.9 (±1.1) a	2.3 (±0.4) a
	CT	13 (±1) b	44.7 (±1.5) a	1.5 (±0.6) b
**2019**		**17 (±4) A**	**44.8 (±1.3) B**	**1.9 (±0.7) A**

^1^ SM: soil management; ^2^ KS: number of kernels per spike; ^3^ TKW: thousand kernel weight; ^4^ NT: no-tillage; ^5^ CT: conventional tillage; means within columns that are followed by the same letter (lowercase letters for SM (a,b); uppercase letters for year (A,B)) are not significantly different at *p* < 0.05.

**Table 9:** Coefficient of determination (R2) and root mean square error (RMSE) between the calculated vegetation indices and the nitrogen content (g m^−2^) within variation.

Vegetation Index	Year	Soil Management	N Content	
			g m^−2^	
			R^2^	RMSE ^1^
ARVI	2018	NT ^2^	0.80 ***	0.61
		CT ^3^	0.08	0.68
	2019	NT	0.73 **	1.48
		CT	0.48 *	1.53
MSAVI2	2018	NT	0.96 ***	0.28
		CT	0.70 **	0.39
	2019	NT	0.84 ***	1.15
		CT	0.42 *	1.61
NDRE	2018	NT	0.88 ***	0.47
		CT	0.59 **	0.45
	2019	NT	0.95 ***	0.62
		CT	0.76 **	0.04
VDVI	2018	NT	0.61 **	0.84
		CT	0.15	0.65
	2019	NT	0.13	1.98
		CT	0.11	2.67
WDRVI	2018	NT	0.78 **	0.64
		CT	0.01	0.71
	2019	NT	0.80 ***	1.28
		CT	0.44 *	1.59
**Mean**	**2018**	**NT**	**0.81**	**0.57**
		**CT**	**0.31**	**0.58**
	**2019**	**NT**	**0.69**	**1.44**
		**CT**	**0.45**	**1.35**

^1^ RMSE: root mean square error; ^2^ NT: no tillage; ^3^ CT: conventional tillage; *: significant at *p* < 0.05%; **: significant at *p* < 0.01%; ***: Significant at *p* < 0.001%.

## References

[1] UN (United Nations) Reports (2017). World Population Prospects: The 2017 Revision.

[2] FAO (Food and Agriculture Organization of the United Nations) (2019). The State of Food Security and Nutrition in the World. Safeguarding against Economic Slowdowns and Downturns.

[3] Pimentel D. (2006). Soil erosion: A food and environmental threat. Environ. Dev. Sustainabil..

[4] Montanarella L., Pennock D.J., McKenzie N., Badraoui M., Chude V., Baptista I., Mamo T., Yemefack M., Singh Aulakh M., Yagi K. (2016). World’s soils are under threat. Soil Sci..

[5] Zhao X., Liu S.L., Pu C., Zhang X.Q., Xue J.F., Ren Y.X., Zhao X.L., Chen F., Lal R., Zhang H.L. (2017). Crop yields under no-till farming in China: A meta-analysis. Eur. J. Agron..

[6] FAO (Food and Agriculture Organization of the United Nations) (2017). The Future of Food and Agriculture: Trends and Challenges.

[7] Foley J.A., Ramankutty N., Brauman K.A., Cassidy E.S., Gerber J.S., Johnston M., Mueller N.D., O’Connell C., Ray D.K., West P.C. (2011). Solutions for a cultivated planet. Nature.

[8] Godfray H.C.J., Garnett T. (2014). Food security and sustainable intensification. Philos. Trans. R. Soc. B.

[9] Tilman D., Balzer C., Hill J., Befort B.L. (2011). Global food demand and the sustainable intensification of agriculture. Proc. Natl. Acad. Sci. USA.

[10] Ashapure A., Jung J., Yeom J., Chang A., Maeda M., Maeda A., Landivar J. (2019). A novel framework to detect conventional tillage and no-tillage cropping system effect on cotton growth and development using multi-temporal UAS data. ISPRS J. Photogramm..

[11] Iocola I., Bassu S., Farina R., Antichi D., Basso B., Bindi M., Dalla Marta A., Danuso F., Doro L., Ferrise R. (2017). Can conservation tillage mitigate climate change impacts in Mediterranean cereal systems? A soil organic carbon assessment using long term experiments. Eur. J. Agron..

[12] Basso B., Ritchie J.T., Sartori L., Grace P.R. (2006). Simulating tillage impacts on soil biophysical properties using the SALUS model. Ital. J. Agron..

[13] Lal R. (1997). Residue management, conservation tillage and soil restoration for mitigating greenhouse effect by CO2-enrichment. Soil Tillage Res..

[14] Lal R. (2004). Managing soil carbon. Science.

[15] Packer I.J., Hamilton G.J. (1993). Soil physical and chemical changes due to tillage and their implications for erosion and productivity. Soil Tillage Res..

[16] López-Garrido R., Madejón E., Murillo J.M., Moreno F. (2011). Short and long-term distribution with depth of soil organic carbon and nutrients under traditional and conservation tillage in a Mediterranean environment (southwest Spain). Soil Use Manag..

[17] Pittelkow C.M., Linquist B.A., Lundy M.E., Liang X., Van Groenigen K.J., Lee J., Van Gestel N., Six J., Venterea R.T., Van Kessel C. (2015). When does no-till yield more? A global meta-analysis. Field. Crops Res..

[18] Triplett G., Dick W.A. (2008). No-tillage crop production: A revolution in agriculture!. Agron. J..

[19] FAO (Food and Agriculture Organization of the United Nations) (2011). Save and Grow: A Policymaker’s Guide for Sustainable Intensification of Small-Holder Agricultural Production.

[20] Tsialtas J.T., Theologidou G.S., Karaoglanidis G.S. (2018). Effects of pyraclostrobin on leaf, leaf physiology, yield and quality of durum wheat under Mediterranean conditions. Crop. Prot..

[21] Hussain J., Khaliq T., Ahmad A., Akhter J., Asseng S. (2018). Wheat responses to climate change and its adaptations: A focus on arid and semi-arid environment. Int. J. Environ. Health Res..

[22] Kahiluoto H., Kaseva J., Balek J., Olesen J.E., Ruiz-Ramos M., Gobin A., Kersebaum K.C., Takáč J., Ruget F., Ferrise R. (2018). Decline in climate resilience of European wheat. Proc. Natl. Acad. Sci. USA.

[23] Oury F.X., Godin C., Mailliard A., Chassin A., Gardet O., Giraud A., Heumez E., Morlais J.Y., Rolland B., Rousset M. (2012). A study of genetic progress due to selection reveals a negative effect of climate change on bread wheat yield in France. Eur. J. Agron..

[24] Hirel B., Tétu T., Lea P.J., Dubois F. (2011). Improving nitrogen use efficiency in crops for sustainable agriculture. Sustainability.

[25] European Parliament (2014). Precision Agriculture: An Opportunity for EU-Farmers—Potential Support with the CAP 2014–2020.

[26] STOA (2016). Precision Agriculture and the Future of Farming in Europe Annex 1: Technical Horizon Scan.

[27] Zhang C., Kovacs J.M. (2012). The application of small unmanned aerial systems for precision agriculture: A review. Precis. Agric..

[28] Pierce F.J., Nowak P. (1999). Aspects of precision agriculture. Adv. Agron..

[29] Davi H., Soudani K., Deckx T., Dufrene E., Le Dantec V., Francois C. (2006). Estimation of forest leaf area index from SPOT imagery using NDVI distribution over forest stands. Int. J. Remote Sens..

[30] Hu J., Su Y., Tan B., Huang D., Yang W., Schull M., Bull M.A., Martonchik J.V., Diner D.J., Knyazikhin Y. (2007). Analysis of the MISR LAI/FPAR product for spatial and temporal coverage, accuracy and consistency. Remote Sens. Environ..

[31] Lamonaca A., Corona P., Barbati A. (2008). Exploring forest structural complexity by multi-scale segmentation of VHR imagery. Remote Sens. Environ..

[32] Pellikka P.K., Lötjönen M., Siljander M., Lens L. (2009). Airborne remote sensing of spatiotemporal change (1955–2004) in indigenous and exotic forest cover in the Taita Hills, Kenya. Int. J. Appl. Earth Observ. Geoinf..

[33] Propastin P., Panferov O. (2013). Retrieval of remotely sensed LAI using Landsat ETM+ data and ground measurements of solar radiation and vegetation structure: Implication of leaf inclination angle. Int. J. Appl. Earth Observ. Geoinf..

[34] Hatfield J.L., Prueger J.H. (2010). Value of Using Different Vegetative Indices to Quantify Agricultural Crop Characteristics at Different Growth Stages under Varying Management Practices. Remote Sens..

[35] Gitelson A.A., Gritz U., Merzlyak M.N. (2003). Relationships between leaf chlorophyll content and spectral reflectance and algorithms for non-destructive chlorophyll assessment in higher plant leaves. J. Plant Physiol..

[36] Huete L., Didan K., Miura T., Rodriguez E.P., Gao X., Ferreira L.G. (2002). Overview of the Radiometric and Biophysical Performance of the MODIS Vegetation Indices. Remote Sens. Environ..

[37] Jordan C.F. (1969). Derivation of Leaf Area Index from Quality of Light on the Forest Floor. Ecology.

[38] Gracia-Romero A., Vergara-Díaz O., Thierfelder C., Cairns J.E., Kefauver S.C., Araus J.L. (2018). Phenotyping conservation agriculture management effects on ground and aerial remote sensing assessments of maize hybrids performance in Zimbabwe. Remote Sens..

[39] Kefauver S.C., Vicente R., Vergara-Díaz O., Fernandez-Gallego J.A., Kerfal S., Lopez A., Melichar J.P.E., Serret Molins M.D., Araus J.L. (2017). Comparative UAV and field phenotyping to assess yield and nitrogen use efficiency in hybrid and conventional barley. Front. Plant Sci..

[40] Asrar G., Fuchs M., Kanemasu E.T., Hatfield J.L. (1984). Estimating absorbed photosynthetic radiation and leaf area index from spectral reflectance in wheat. Agron. J..

[41] Nguy-Robertson A.L., Peng Y., Gitelson A.A., Arkebauer T.J., Pimstein A., Herrmann I., Karnieli A., Rundquist D.C., Bonfil D.J. (2014). Agricultural and forest meteorology estimating green LAI in four crops: Potential of determining optimal spectral bands for a universal algorithm. Agric. For. Meteorol..

[42] Gamon J.A., Huemmrich K.F., Wong C.Y.S., Ensminger I., Garrity S., Hollinger D.Y., Noormets A., Peñuelas J. (2016). A remotely sensed pigment index reveals photosynthetic phenology in evergreen conifers. Proc. Natl. Acad. Sci. USA.

[43] Gitelson A.A., Viña A., Ciganda V., Rundquist D.C., Arkebauer T.J. (2005). Remote estimation of canopy chlorophyll content in crops. Geophys. Res. Lett..

[44] Haboudane D., Tremblay N., Miller J.R., Vigneault P. (2008). Remote estimation of crop chlorophyll content using spectral indices derived from hyperspectral data IEEE Trans. Geosci. Remote Sens..

[45] Herrmann I., Karnieli A., Bonfil D.J., Cohen Y., Alchanatis V. (2010). SWIR-based spectral indices for assessing nitrogen content in potato fields. Int. J. Remote Sens..

[46] Feng W., Yao X., Zhu Y., Tian Y.C., Cao W.X. (2008). Monitoring leaf nitrogen status with hyperspectral reflectance in wheat. Eur. J. Agron..

[47] Costa J.M., Grant O.M., Chaves M.M. (2013). Thermography to explore plant-environment interactions. J. Exp. Bot..

[48] Yousfi S., Kellas N., Saidi L., Benlakehal Z., Chaou L., Siad D., Herda F., Karrou M., Vergara O., Gracia A. (2016). Comparative performance of remote sensing methods in assessing wheat performance under Mediterranean conditions. Agric. Water Manag..

[49] Khanal S., Fulton J., Shearer S. (2017). An overview of current and potential applications of thermal remote sensing in precision agriculture. Comput. Electron. Agric..

[50] Moran M.S., Inoue Y., Barnes E.M. (1997). Opportunities and limitations for image-based remote sensing in precision crop management. Remote Sens. Environ..

[51] Seelan S.K., Laguette S., Casady G.M., Seielstad G. (2003). Remote sensing applications for precision agriculture: A learning community approach. Remote Sens. Environ..

[52] Scudiero E., Teatini P., Corwin D.L., Deiana R., Berti A., Morari F. (2013). Delineation of site-specific management units in a saline region at the Venice Lagoon margin, Italy, using soil reflectance and apparent electrical conductivity. Comput. Electron. Agric..

[53] Corwin D.L., Lesch S.M. (2005). Apparent soil electrical conductivity measurements in agriculture. Comput. Electron. Agric..

[54] Yin R., Eisenhauer N., Schmidt A., Gruss I., Purahong W., Siebert J., Schädler M. (2019). Climate change does not alter land-use effects on soil fauna communities. Appl. Soil Ecol..

[55] Thakur M.P., Reich P.B., Hobbie S.E., Stefanski A., Rich R., Rice K.E., Eddy W.C., Eisenhauer N. (2018). Reduced feeding activity of soil detritivores under warmer and drier conditions. Nat. Clim. Chang..

[56] Eastwood C.R., Kenny S. (2009). Art or science? Heuristic versus data driven grazing management on dairy farms. J. Farm. Syst. Res. Ext..

[57] Nuthall P.L. (2012). The intuitive world of farmers—The case of grazing management systems and experts. Agric. Syst..

[58] Eastwood C.R., Jago J.G., Edwards J.P., Burke J.K. (2016). Getting the most out of advanced farm management technologies: Roles of technology suppliers and dairy industry organizations in supporting precision dairy farmers. Anim. Prod. Sci..

[59] Bewley J.M., Russell R.A. Reasons for slow adoption rates of precision dairy farming technologies: Evidence from a producer survey. Proceedings of the First North American Conference on Precision Dairy Management.

[60] Hoes A.C., Beekman V., Regeer B.J., Bunders J.F.G. (2012). Unravelling the dynamics of adopting novel technologies: An account of how the closed greenhouse opened-up. Int. J. Foresight Innov. Policy.

[61] García-Palacios P., Maestre F.T., Kattge J., Wall D.H. (2013). Climate and litter quality differently modulate the effects of soil fauna on litter decomposition across biomes. Ecol. Lett..

[62] Wall D.H., Bradford M.A., St. John M.G., Trofymow J.A., Behan-Pelletier V., Bignell D.E., Dangerfield J.M., Parton W.J., Rusek J., Voigt W. (2008). Global decomposition experiment shows soil animal impacts on decomposition are climate-dependent. Glob. Chang. Biol..

[63] FAO (Food and Agriculture Organization of the United Nations) (2006). World Soil Resources Report 103.

[64] Hargreaves G.H., Samani Z.A. (1982). Estimating potential evapotranspiration. J. Irrig. Drain. Engr..

[65] FAO (Food and Agriculture Organization of the United Nations) (1998). Crop Evapotranspiration—Guidelines for Computing Crop Water Requirements—FAO Irrigation and Drainage Paper 56.

[66] Orsini R., Basili D., Belletti M., Bentivoglio D., Bozzi C.A., Chiappini S., Conti C., Fiorentini M., Galli A., Giorgini E. (2019). Setting of a precision farming robotic laboratory for cropping system sustainability and food safety and security: Preliminary results. IOP Conference Series: Earth and Environmental Science.

[67] Seddaiu G., Iocola I., Farina R., Orsini R., Iezzi G., Roggero P.P. (2016). Long-term effects of tillage practices and N fertilization in rainfed Mediterranean cropping systems: Durum wheat, sunflower and maize grain yield. Eur. J. Agron..

[68] Zadoks J.C., Chang T.T., Konzak C.F. (1974). A decimal code for the growth stages of cereals. Weed Res..

[69] Dumas A. (1826). Ann. Chim..

[70] Buckee G.K. (1994). Determination of total nitrogen in Barley, Malt and Beer by Kjeldahl procedures and the Dumas combustion method. J. Inorg. Biochem..

[71] Verhoeven G. (2011). Taking computer vision aloft archaeological three-dimensional reconstructions from aerial photographs with photoscan. Archaeol. Prospect..

[72] Xue J., Su B. (2017). Significant Remote Sensing Vegetation Indeces: A Review of Developments and Applications. J. Sens..

[73] Korhonen L., Ali-Sisto D., Tokola T. (2015). Tropical forest canopy cover estimation using satellite imagery and airborne lidar reference data. Silva Fenn..

[74] Leprieur C., Kerr Y.H., Mastorchio S., Meunier J.C. (2000). Monitoring vegetation cover across semi-arid regions: Comparison of remote observations from various scales. Int. J. Remote Sens..

[75] Barnes E.M., Clarke T.R., Richards S.E., Colaizzi P.D., Haberland J., Kostrzewski M., Waller P., Robert P.C., Rust R.H., Larson W.E. (2000). Coincident detection of crop water stress, nitrogen status and canopy density using ground based multispectral data. Proceedings of the Fifth International Conference on Precision Agriculture.

[76] Wang X., Wang M., Wang S., Wu Y. (2015). Extraction of vegetation information from visible unmanned aerial vehicle images. Nongye Gongcheng Xuebao/Trans. Chin. Soc. Agric. Engin..

[77] Gitelson A.A. (2004). Wide dynamic range vegetation index for remote quantification of biophysical characteristics of vegetation. J. Plant Physiol..

[78] Ercoli L., Masoni A., Mariotti M., Pampana S., Pellegrino E., Arduini I. (2017). Effect of preceding crop on the agronomic and economic performance of durum wheat in the transition from conventional to reduced tillage. Eur. J. Agron..

[79] Giunta F., Pruneddu G., Motzo R. (2019). Grain yield and grain protein of old and modern durum wheat cultivars grown under different cropping systems. Field Crop. Res..

[80] Orsini R., De Sanctis G., Toderi M., Perugini M., Roggero P.P. (2008). Nitrate concentration of Runoff in Two Rural Micro—Catchments of Central Italy: Results from a Ten-Years Survey. Ital. J. Agron..

[81] Badalíková B. (2010). Influence of Soil Tillage on Soil Compaction. Soil Eng. Soil Biol..

[82] Gozubuyuk Z., Sahin U., Ozturk I., Çelik A., Adiguzel M.C. (2014). Tillage effects on certain physical and hydraulic properties of a loamy soil under a crop rotation in a semi-arid region with a cool climate. Catena.

[83] Gozubuyuk Z., Sahin U., Adiguzel M.C., Ozturk I., Celik A. (2015). The influence of different tillage practices on water content of soil and crop yield in vetch–winter wheat rotation compared to fallow–winter wheat rotation in a high altitude and cool climate. Agric. Water Manag..

[84] Bellotti B., Rochecouste J.F. (2014). The development of Conservation Agriculture in Australia-Farmers as innovators. Int. Soil Water Conserv. Res..

[85] Page K.L., Dang Y., Dalal R.C. (2013). Impacts of conservation tillage on soil quality, including soil-borne crop diseases, with a focus on semi-arid grain cropping systems. Aust. Plant Pathol..

[86] Pittelkow C., Liang X., Linquist B.A., Van Groenigen K.J.L., Lee J., Lundy M.E., Van Gestel N., Six J., Venterea R.T., Van Kessel C. (2015). Productivity limits and potentials of the principles of conservation agriculture. Nature.

[87] Chan K.Y., Roberts W.P., Heenan D.P. (1992). Organic carbon and associated properties of a red earth after 10 years rotation under different stubble and tillage practices. Aust. J. Soil Res..

[88] González-Chávez C.A., Aitkenhead-Peterson J.A., Gentry T.J., Zuberer D., Hons F., Loeppert R. (2010). Soil microbial community, C, N, and P responses to long-term tillage and crop rotation. Soil Tillage Res..

[89] Redel Y.D., Rubio R., Rouanet J.L., Borie F. (2007). Phosphorus bioavailability affected by tillage and crop rotation on a Chilean volcanic derived Ultisol. Geoderma.

[90] Fuentes J.P., Flury M., Huggins D.R., Bezdicek D.F. (2003). Soil water and nitrogen dynamics in dryland cropping systems of Washington State U.S.A. Soil Tillage Res..

[91] Kassam A., Friedrich T., Derpsch R., Lahmar R., Mrabet R., Basch G., González-Sánchez E.J., Serraj R., Bash G., González S.E.J. (2012). Conservation agriculture in the dry Mediterranean climate. Field Crop. Res..

[92] Jemai I., Ben Aissa N., Ben Guirat S., Ben-Hammouda M., Gallali T. (2013). Impact of three and seven years of no-tillage on the soil water storage, in the plant root zone, under a dry subhumid Tunisian climate. Soil Tillage Res..

[93] Mohammadi M., Karimizadeh R., Shafazadeh M.K., Sadeghzadeh B. (2013). Statistical analysis of durum wheat yield under semi-warm dryland condition. Aust. J. Crop. Sci..

[94] Labuchagne M.T., Elago O., Koen E. (2009). The influence of temperature extremes on some quality and starch characteristics in bread, biscuit and durum wheat. J. Cereal Sci..

[95] Orsini R., Fiorentini M., Zenobi S. Testing vegetation index categories as influenced by soil management and nitrogen fertilization in cereal based cropping systems. Proceedings of the 2019 International IEEE Workshop on Metrology for Agriculture and Foresty.

[96] Fiorentini M., Zenobi S., Giorgini E., Basili D., Conti C., Pro C., Monaci E., Orsini R. (2019). Nitrogen and chlorophyll status determination in durum wheat as influenced by fertilization and soil management: Preliminary results. PLoS ONE.

[97] Stagnari F., Ramazzotti S., Pisante M., Lichtfouse E. (2009). Conservation Agriculture: A Different Approach for Crop Production through Sustainable Soil and Water Management: A Review. Organic Farming, Pest Control and Remediation of Soil Pollutants.

[98] Barut Z.B., Ertekin C., Ali Karaaga H. (2011). Tillage effects on energy use for corn silage in Mediterranean Coastal of Turkey. Energy.

[99] Moussa-Machraoui S.B., Errouissi F., Ben-Hammouda M., Nouira S. (2010). Comparative effects of conventional and no-tillage management on some soil properties under Mediterranean semi-arid conditions in north-western Tunisia. Soil Tillage Res..

[100] De Sanctis G., Roggero P.P., Seddaiu G., Orsini R., Porter C.H., Jones J.W. (2012). Long-term no tillage increased soil organic carbon content of rain-fed cereal systems in a Mediterranean area. Eur. J. Agron..

[101] Avio L., Castaldini M., Fabiani A., Bedini S., Sbrana C., Turrini A., Giovannetti M. (2013). Impact of nitrogen fertilization and soil tillage on arbuscular mycorrhizal fungal communities in a Mediterranean agroecosystem. Soil Biol. Biochem..

[102] Pastorelli R., Vignozzi N., Landi S., Piccolo R., Orsini R., Seddaiu G., Roggero P.P., Pagliai M. (2013). Consequences on macroporosity and bacterial diversity of adopting a no-tillage farming system in a clayish soil of Central Italy. Soil Biol. Biochem..

[103] De Vita P., Di Paolo E., Fecondo G., Di Fonzo N., Pisante M. (2007). No-tillage and conventional tillage effects on durum wheat yield, grain quality and soil moisture in southern Italy. Soil Tillage Res..

[104] López-Bellido L., Muñoz-Romero V., López-Bellido R.J. (2013). Nitrate accumulation in the soil profile 564 Long-term effects of tillage, rotation and N rate in a Mediterranean Vertisol. Soil Tillage Res..

